# The impact of early detection and intervention of breast cancer‐related lymphedema: a systematic review

**DOI:** 10.1002/cam4.691

**Published:** 2016-03-19

**Authors:** Chirag Shah, Douglas W. Arthur, David Wazer, Atif Khan, Sheila Ridner, Frank Vicini

**Affiliations:** ^1^ Department of Radiation OncologyTaussig Cancer InstituteCleveland ClinicClevelandOhio; ^2^ Department of Radiation OncologyMassey Cancer Center, Virginia Commonwealth UniversityRichmond, Virginia; ^3^ Department of Radiation OncologyTufts University School of MedicineBostonMassachusetts; ^4^ Department of Radiation OncologyBrown UniversityProvidenceRhode Island; ^5^ Department of Radiation OncologyRobert Wood Johnson Medical SchoolRutgers UniversityNew BrunswickNew Jersey; ^6^ School of NursingVanderbilt UniversityNashvilleTennessee; ^7^ Michigan Healthcare Professionals/21^st^ Century OncologyFarmington HillsMichigan

**Keywords:** Breast cancer, detection, intervention, lymphedema, prevention

## Abstract

Breast cancer‐related lymphedema (BCRL) has become an increasingly important clinical issue as noted by the recent update of the 2015 NCCN breast cancer guidelines which recommends to “educate, monitor, and refer for lymphedema management.” The purpose of this review was to examine the literature regarding early detection and management of BCRL in order to (1) better characterize the benefit of proactive surveillance and intervention, (2) clarify the optimal monitoring techniques, and (3) help better define patient groups most likely to benefit from surveillance programs. A Medline search was conducted for the years 1992–2015 to identify articles addressing early detection and management of BCRL. After an initial search, 127 articles were identified, with 13 of these studies focused on early intervention (three randomized (level of evidence 1), four prospective (level of evidence 2–3), six retrospective trials (level of evidence 4)). Data from two, small (*n* = 185 cases), randomized trials with limited follow‐up demonstrated a benefit to early intervention (physiotherapy, manual lymphatic drainage) with regard to reducing the rate of chronic BCRL (>50% reduction) with two additional studies underway (*n* = 1280). These findings were confirmed by larger prospective and retrospective series. Several studies were identified that demonstrate that newer diagnostic modalities (bioimpedance spectroscopy, perometry) have increased sensitivity allowing for the earlier detection of BCRL. Current data support the development of surveillance programs geared toward the early detection and management of BCRL in part due to newer, more sensitive diagnostic modalities.

## Introduction

The number of long‐term breast cancer survivors continues to increase secondary to improved outcomes over the past several decades 1, 2. This growing population of patients has resulted in an increased focus on survivorship and the management and prevention of acute and chronic toxicities. One such concern is breast cancer‐related lymphedema (BCRL). BCRL can have a significant, detrimental impact on the quality of life of breast cancer survivors. With the increased use of multimodality therapy including surgery, local‐regional radiation therapy, and certain systemic chemotherapeutic agents, the number of cases of BCRL may continue to increase. Due to the heterogeneity of measurement techniques and a lack of consistent definitions for BCRL, limited data are available quantifying the rates of BCRL (based upon locoregional and systemic therapies) with ranges from 5% with breast‐conserving surgery alone to over 50% with mastectomy, postmastectomy radiation with regional irradiation, and chemotherapy 3, 4. Despite reductions in the rate of BCRL with newer surgical techniques (SLN biopsy), recent data still demonstrate rates of 10–30% at 2 years 5.

BCRL can lead to devastating complications including infections as well deterioration in quality of life through a reduction in function of the affected limb 6, 7, 8. However, close examination of the pathophysiology behind BCRL demonstrates that there is an acute and chronic phase to the process. In the early phase of BCRL, the accumulation of fluid may not be clinically apparent with subclinical volume accumulation (Stage 0 or latent stage) 9. This can progress to clinically evident lymphedema that resolves with elevation of the limb (Stage I) and can be characterized by a lack of fibrosis 9. Subsequently, the chronic, irreversible phase of the disease can be classified by the development of intradermal fibrosis 9. Since there is a reversible subclinical phase, the potential exists for detection and treatment of BCRL at this phase of the process. However, older diagnostic tests have limited ability to detect subclinical BCRL reducing the potential for early detection and therefore early intervention 9, 10, 11.

In 2015, the NCCN Breast Cancer Panel adopted a new standard recommendation for the follow‐up care and monitoring of breast cancer survivors, specifically relating to BCRL. The task force recommended “to educate, monitor, and refer for lymphedema management” as part of standard follow‐up 12. Given this challenge and the paucity of data establishing standard guidelines to help monitor for BCRL (and when to intervene), the purpose of this review was to examine the literature on the role of early diagnosis and management of BCRL in women treated for breast cancer. The goal is to provide an objective assessment of the literature in order to allow clinicians information on how to better apply recent information on early detection and intervention of BCRL for use in patient follow‐up care.

## Methods

This systematic review was conducted in line with the Preferred Reporting Items for Systematic Reviews and Meta‐Analyses (PRISMA) guidelines 13; no review protocol exists for this review. While not part of the systematic review, 20 studies were identified that evaluated traditional and new diagnostic modalities for BCRL. Eligibility criteria included published studies in English evaluating patients treated for breast cancer with some form of early lymphedema intervention and/or diagnostic assessment between 1990 and 2015. The required information included the number of patients, length of follow‐up, intervention (if present), procedures for lymphedema assessment/documentation, timing of assessment, and outcomes. No minimum length of follow‐up was required for the initial search. A 25 year interval of publication was chosen in order to focus on more recent literature, which included contemporary surgical, radiation, and systemic therapy modalities. Sources of information for the review included Medline/PubMed, those found in references from the major articles identified and articles known to the authors.

The following search terms were used to search the Medline database: breast cancer, lymphedema, early management, subclinical diagnosis, and prospective management. A single physician (CS) performed the following searches: BCRL (*n* = 303), breast cancer/lymphedema/detection (*n* = 51), breast cancer/lymphedema/early (*n* = 200), breast cancer/lymphedema/prospective (*n* = 267), breast cancer/lymphedema/subclinical (*n* = 7). A final more extensive search using breast cancer and lymphedema (*n* = 1510) was performed to ensure completeness of the search. Based on the initial searches, a total of 127 articles examining early intervention were selected for screening including 10 articles that were known to the authors. With regard to screening the articles, when multiple updates from a single institutional series were available, the most recent data were utilized with the exception of data that was published in an older publication and not updated in subsequent series. Articles were evaluated independently by a single physician and data was extracted including the type of study (prospective vs. retrospective), institution, number of patients, follow‐up, interventions utilized/technique, assessment methods and procedure, and documented outcomes. Studies were excluded due to lack of clear lymphedema assessment/intervention, unclear outcomes, or outcomes presented that were not within the scope of the review. Bias for each study was evaluated by examining assessment techniques utilized (observer bias in nonblinded studies), conflicts of interest, and statistical analyses utilized. Due to the differences in assessment techniques, timing of measurements, and interventions utilized, data were unable to be pooled for this review. Of the articles identified, 13 studies were found to meet the eligibility criteria and the scope of the review. Of these studies, three (23%, level 1 evidence) were randomized trials, four (31%, level 2–3 evidence) were prospective studies, and six (46%, level 4 evidence) were retrospective studies.

## Results

Prior to reviewing literature evaluating the impact of early detection/intervention for BCRL, the techniques available to diagnose BCRL need to be reviewed. Traditionally, the diagnosis of BCRL has been based on several techniques including circumferential arm measurements, water displacement, and patient surveys 9, 10, 11. Despite the frequent utilization of these techniques, their widespread and consistent application has been limited secondary to issues concerning low sensitivity, large interobserver variability, and a lack of standardization and diagnostic cutoffs. Collectively, these concerns seriously limit the ability for their use in the early detection and intevention of BCRL 9, 14, 15, 16, 17, 18, 19.

Diagnostic modalities for BCRL have continued to evolve with newer techniques available that have increased sensitivity, allowing for the subclinical detection of BCRL and therefore, early intervention. One such technique is optoelectronic perometry, which uses infrared light to measure limb volume; prospective data have demonstrated the feasibility of the technique and the ability for subclinical detection of BCRL. However, limitations with perometry include space requirements and difficulties associated with its application 13, 20, 21, 22. Dual‐energy X‐ray absorptiometry is another technique that has demonstrated improved consistency compared with circumferential arm measurements, 23 although limited data are available beyond a handful of small series and further, it uses low‐level X‐rays, exposing patients to radiation. A third modality that has been increasingly utilized is bioimpedance spectroscopy (BIS). BIS uses electrical current to detect the volume of the extracellular space that can then be converted into a measurement index with a validated cutoff. Multiple studies have documented the feasibility of implementing BIS in the clinic along with its increased sensitivity (compared with traditional techniques—circumference measurements, patient survey) as well as its ability to detect the subclinical phase of the disease (up to 10 months prior to the appearance of clinical symptoms in some studies) 24, 25, 26, 27, 28, 29. Taken together, these data demonstrate that new diagnostic modalities have increased sensitivity and the ability to detect subclinical BCRL. Further, the feasibility of implementing BCRL programs applying these diagnostic techniques, allowing for early detection and intervention, has been established 20, 26, 27.

### Review of early management literature

A comprehensive review of all the available literature demonstrated increasing amounts of data supporting the benefits of early intervention in BCRL; however, these studies were generally limited by (1) small patient numbers, (2) lack of formal statistical randomization, and (3) a lack of detail regarding methodology.

### Randomized trials (early intervention)

Three randomized trials evaluating the role of early detection and intervention were identified as well as a fourth, recently opened clinical trial. A randomized study from Madrid enrolled 120 women treated with axillary lymph node dissection (ALND). Patients were randomized to either (1) a program of manual lymphatic drainage (MLD), massage, and exercise (along with BCRL education) or (2) BCRL education alone. Patients were followed at 3, 6, and 12 months after surgery; BCRL was analyzed using the circumferential measurement technique and BCRL was defined as a 2 cm or greater increase at any two adjacent points compared with the unaffected limb. At 1 year, 25% of patients randomized to education alone developed BCRL as compared with 7% in the MLD/massage/exercise arm of the trial (HR 0.28, *P* = 0.01) 30. Despite demonstrating a significant difference in the rate of development of BCRL, limitations of the study included the use of a low‐sensitivity diagnostic technique, a small number of patients resulting in limited statistical power, short follow‐up, and a failure to provide data on factors associated with BCRL development between treatment arms.

Another randomized trial was identified from the University of Queensland. This study randomized 65 women following axillary dissection to prospective monitoring and treatment with physiotherapy or to surveillance alone. BCRL was evaluated prior to surgery and at regular postoperative intervals (day 5, 1/2/6/12/24 months) using both circumferential arm measurements and with BIS. For the study, BCRL was defined as a 200 cc/1.5 cm increase in volume as compared with the untreated arm. At 2 years, the incidence of BCRL was 11% with early intervention as compared with 30% with surveillance and no intervention 31. Strengths of the study include the use of a high‐sensitivity diagnostic technique and blinded assessments. Limitations included a small number of participants and differences in factors between the patients in the two arms that may impact outcome (i.e., number of nodes sampled, wound infections, radiation therapy receipt).

An ongoing randomized trial from the University of Sydney is evaluating the role of early intervention with exercise and surveillance (weekly) as compared with standard care (pamphlets, visiting a physiotherapist at the time of surgery). The trial calls for enrollment of 180 women with early‐stage breast cancer with BCRL assessments at baseline, 8 weeks, and 6 months. BCRL will be assessed via BIS with blinded measurements and stratification by axillary intervention 32. Finally, a recently opened large randomized study is evaluating early detection and intervention utilizing BIS as compared to standard circumferential arm measurements. This multi‐institutional study (sponsored by Vanderbilt University) has a targeted accrual of 1100 patients and provides for 3‐years of follow‐up; eligibility criteria for the trial focus on patients at higher risk for BCRL and randomizes patients to circumferential arm measurements or BIS. Patients on the BIS arm of the trial that exceed an increase in 10 (L‐Dex score) will undergo circumferential arm measurements and short‐course treatment with a compression sleeve (22–32 mm Hg) and gauntlet with similar intervention for patients in the circumference arm experiencing a 5–10% increase in volume. Table 1 summarizes the randomized trials published to date focusing on early intervention. Including the currently enrolling trials, a total of 1465 patients will have been enrolled with outcomes available on only 185 to date.

**Table 1:** Studies addressing early diagnosis/management of breast cancer‐related lymphedema

Institution	Year	Number of patients	Diagnostic technique	Intervention	Results
Randomized					
Alcala de Heneres University, Madrid 30	2010	120 (ALND)	Circumference	Education versus Early physiotherapy	Early physiotherapy reduced BCRL at 1 year (7% vs. 25%, *P* = 0.01)
University of Queensland 31	2002	65 (ALND)	Circumference / Bioimpedance	Surveillance versus Early physiotherapy	Early physiotherapy reduced BCRL at two years (11% vs. 30%)
Prospective					
National Naval Medical Center 21, 33	2008	196 (43 treated)	Perometry	Prospective surveillance program and compression garment use	Early treatment led to 48 cc volume decrease with mean 4.4 week use; 5 year update 25% subclinical lymphedema, 6% advanced lymphedema
University of Pittsburgh 35	2014	186	Bioimpedance	Prospective surveillance program and compression garment use	33% of patients developed subclinical BCRL, 4.4% progress to clinical BCRL
Istanbul Ilim University 37	2015	37	Bioimpedance/Clinical	Prospective surveillance program	22% developed BCRL with only bioimpedance able to detect patients with subclinical BCRL
University Hospital of South Manchester 38	2015	964	Bioimpedance/Perometry	Prospective surveillance program	Bioimpedance detected a greater number of BCRL cases, threshold for early intervention 5–10%
Retrospective					
University of New Mexico 39	1999	69	Circumference	–	Initial BCRL volume correlated with response to treatment
University of Pennsylvania 40	2010	1713	Circumference	–	Patients with low‐volume BCRL (0.5–2.0 cm increase), low rate of progression to advanced BCRL
Lund University 42	2010	292	Water Displacement	–	Patients with low‐volume BCRL at diagnosis were less likely to develop chronic large volume increase (16% vs. 10%)

ALND, axillary lymph node dissection; BCRL, breast cancer‐related lymphedema.

### Prospective and retrospective data

Data supporting early intervention also comes from a prospective trial of 196 women who underwent pre‐ and postoperative (1, 3, 6, 12, 18 months) perometry measurements using a 3% threshold as the diagnostic criteria for BCRL. Of the initial 196 patients, 43 were diagnosed with BCRL and treated with a 20–30 mmHg compression garment. Early intervention was found to reduce arm volumes while limiting the need for further more aggressive therapies 21. The 5‐year update of this study demonstrated a 25% rate of subclinical lymphedema (>3% increase) with a 5.6% rate of advanced lymphedema (Stage I/II). These findings were felt to support the concept that a prospective surveillance model can reduce rates of chronic BCRL 33. While this study utilized a more sensitive technique, it was limited by the lack of randomization, and the small number of patients that progressed (*n* = 43) represented the intervention subgroup. A retrospective analysis of 46 patients from the same group found that when measuring BCRL with perometry (preoperatively, 1/2/6/9/12 months postoperatively), segmental changes developed prior to clinical diagnosis of BCRL and allowed for the subclinical diagnosis of BCRL 34. These findings were consistent with data from Soran et al., (also a prospective study) that enrolled 186 patients who underwent BIS measurements every 3–6 months for 5 years. Patients diagnosed with subclinical BCRL underwent short‐term physical therapy, education, and were provided compression sleeves. A total of 33% of patients were diagnosed with subclinical BCRL with only 4.4% developing clinical BCRL 35. Subset analysis from the program also demonstrated that risk calculators for BCRL (Cleveland Clinic calculator) were not accurate in predicting BCRL when utilizing diagnostic techniques that can detect subclinical BCRL 36. Similarly, a small prospective study of 37 patients from Turkey evaluated the development of BCRL using BIS as well as clinical measurements and found BIS was able to detect cases of subclinical BCRL beyond clinical measurements using a 3 month follow‐up schedule for the first year following treatment 37. Finally, one of the largest prospective studies comes from Manchester; this multicenter study enrolled 964 patients (612 with minimum 6 months of follow‐up) with patients undergoing BIS and perometer measurements including preoperative assessment as well as routine postoperative measurement. BIS detected more patients with BCRL (53 vs. 31) and the threshold for early intervention was felt to be a 5–10% volume increase which was more predictive than clinical symptoms 38.

Further validation of the concepts of early detection and intervention come from retrospective data evaluating outcomes based on initial BCRL volume. Ramos et al. presented a retrospective series of 69 women treated for BCRL with diagnosis and follow up measurements made via circumferential arm measurements. A significant difference in BCRL response rates to treatment was noted based on initial arm volume with a mean reduction of 78% in patients with less than 250 cc of initial edema as compared to 56% for patients presenting with 250–500 cc of edema and 38% for patients with greater than 500 cc of edema 39. A large retrospective series of 1713 patients with Stage I/II breast cancer validated these findings noting that in patients with small volume increases at presentation (0.5–2.0 cm increase), 80% did not progress to more advanced BCRL at 1 year. Factors associated with progression in mild BCRL cases were BMI greater than 35, one or more positive lymph nodes, and supraclavicular irradiation 40. An update of this study demonstrated consistent findings with a third of patients progressing to more severe lymphedema at 5 years of follow‐up. The authors recommended routine arm measurements after treatment to allow for earlier diagnosis and intervention 41. It should be noted that while this study started with a large cohort of patients, only 109 subsequently developed BCRL and represented the focus of the analysis. Also, measurements were only taken when patients had clinically evident changes in arm circumference; so, no subclinical detection and intervention was employed.

A retrospective analysis from Sweden evaluated 292 breast cancer patients who underwent ALND as well as postoperative radiation to the breast and axilla with BCRL assessed via water displacement twice yearly for 10 years. Results demonstrated that patients diagnosed with low‐volume BCRL (5–10% increase in volume) were less likely to develop chronic large arm volume increases (greater than 20%) compared to patients diagnosed with larger initial volumes (10.1% vs. 15.8%) 42. While this study provides one of the only series with 10‐year follow‐up, it utilized a less sensitive technique (water circumference) and did not control substantially for interventions utilized. Table 1 summarizes the nonprospective studies reviewed; while these studies are larger, they are limited by their retrospective nature and the inability to control for factors such as weight, and factors associated with BCRL (chemotherapy, radiation therapy). Based on the publications reviewed, there currently exists data supporting early intervention.

### Formal guidelines for BCRL surveillance

Although this review identified substantial literature supporting the early detection and treatment of BCRL, many questions still remain. These questions include (1) defining appropriate surveillance programs with respect to frequency of testing and/or optimal methods to employ, and (2) establishing patient groups that derive the greatest benefit from proactive monitoring. Fortunately, literature addressing guidelines was identified from multiple organizations helping to establish appropriate programs for the early detection and management of BCRL. As discussed previously, the NCCN has released updated breast cancer guidelines for 2015 that identify the importance of BCRL and recommend education, monitoring, and treatment as standard follow‐up procedures 12. These recommendations are consistent with the findings from this review 12. Guidelines from the United Kingdom have also been released and highlight the high sensitivity and specificity of new diagnostic modalities such as multifrequency bioimpedance 43. Further, the Avon Foundation for Women White Paper on BCRL suggests that early physiotherapy may be an effective early intervention and further supports the use of new modalities for early detection as a means to identify patients suitable for early intervention 44. The position paper suggests that while perometry and BIS are good options, that the “more economical alternative of BIS holds great promise as an aid in the early detection of LE. 44 ^”^ Finally, data from the National Lymphedema Network suggests that new modalities of diagnosis (perometry, BIS) may be utilized as an adjunct to traditional therapies or in lieu of them for early detection 45. These evidence‐based guidelines highlight the increasing data available on new diagnostic modalities and their value in the early detection of BCRL.

### Identifying a high‐risk cohort and model

Multiple models have been developed with regard to prospective surveillance and intervention for BCRL utilizing newer diagnostic modalities including perometry and BIS 20, 22, 26. However, no comparison of models has been made and future work is required to identify the best schedule with respect to early identification while minimizing patient visits and costs. With respect to duration of follow‐up, data support surveillance for at least 5 years following treatment with recent data showing that 89% of BCRL diagnoses occur within the first 3 years following treatment 40, 41, 46. While various testing frequencies have been used, one potential schedule often quoted is to obtain preoperative and perioperative measurements followed by postoperative measurements at 3, 6, 12, 18, 24, and 36 months following treatment 47. Ongoing prospective surveillance programs should be helpful to further clarify these issues in the years to come.

A major concern with surveillance programs is identifying which patients should be included, as it is not feasible or cost‐effective to enroll all breast cancer patients. However, based on this review (and specifically evaluating the incidence of BCRL), high‐risk patients may include (1) those undergoing ALND, (2) regional nodal irradiation, and/or (3) taxane‐based chemotherapy 48, 49, 50, 51. Similarly, data have demonstrated a high risk of significant BCRL in patients with increased BMI and/or a history of cellulitis, both of which may be included into surveillance inclusion criteria 52. Models have been created to identify those at highest risk for developing BCRL, but as noted above, the models have not correlated well with prospective data 36, 48.

## Discussion

The purpose of this review was to examine data focusing on early intervention and new diagnostic modalities that allow for the early detection of BCRL. A key finding of our study is that to date, two randomized trials have been performed supporting early intervention with physiotherapy/exercise/MLD (although both studies were small and had short follow‐up). Future studies have been planned to increase the number of patients investigated and to utilize the newer diagnostic modalities supported by nonrandomized prospective series and retrospective data. However, additional data from early intervention protocols should be prospectively collected to help clarify the magnitude and extent of their benefit on reducing chronic BCRL and improving quality of life 20, 22, 26. Further, new diagnostic modalities offer the ability to provide clinicians with standardized objective cut points to initiate therapy, something that has been previously limited with older techniques.

This review did not attempt to examine the costs associated with early intervention and detection or its overall cost‐efficacy. However, an economic analysis from Shih et al. found that women with BCRL had significantly higher overall medical costs in the 2 years following treatment ($23, 167 vs. $14,877) 53. Therefore, future studies examining the cost of such programs most not only factor in the cost of detection equipment and treatment but also the long‐term savings gained by prevention of chronic sequelae of BCRL. A recent analysis compared a prospective surveillance model with traditional “impairment‐based care” and found that while the cost was estimated to be $636 per year to place patients in the prospective surveillance model, for patients using the traditional model, the cost was $3,124 per year to manage BCRL 54. Future studies examining early intervention and detection should include cost analyses in order to determine if these strategies not only improve clinical outcomes but also represent a cost‐effective approach.

## Conflicts of Interest

Chirag Shah, MD, Douglas W. Arthur, MD and Frank A. Vicini, MD—Research Advisory Committee for Impedimed.
